# Increased Receptor Affinity and Reduced Recognition by Specific Antibodies Contribute to Immune Escape of SARS-CoV-2 Variant Omicron

**DOI:** 10.3390/vaccines10050743

**Published:** 2022-05-09

**Authors:** Anne-Cathrine S. Vogt, Gilles Augusto, Byron Martina, Xinyue Chang, Gheyath Nasrallah, Daniel E. Speiser, Monique Vogel, Martin F. Bachmann, Mona O. Mohsen

**Affiliations:** 1 Department of BioMedical Research, University of Bern, 3010 Bern, Switzerland; gilles.sousaaugusto@dbmr.unibe.ch (G.A.); xinyue.chang@dbmr.unibe.ch (X.C.); daniel.speiser@unil.ch (D.E.S.); monique.vogel@dbmr.unibe.ch (M.V.); martin.bachmann@dbmr.unibe.ch (M.F.B.); 2 Department of Immunology RI, University Hospital Bern, 3010 Bern, Switzerland; 3 The Jenner Institute, University of Oxford, Oxford OX3 7DQ, UK; 4 Department of Viroscience, Erasmus Medical Center, 3015 Rotterdam, The Netherlands; b.martina@protinhi.com; 5 Artemis Bio-Support, 2629 Delft, The Netherlands; 6 Biomedical Research Complex, Qatar University, Doha P.O. Box 2713, Qatar; gheyath.nasrallah@qu.edu.qa; 7 Department of Biomedical Science, College of Health Sciences, Member of QU Health, Qatar University, Doha P.O. Box 2713, Qatar; 8 Saiba GmbH, 8808 Pfaeffikon, Switzerland

**Keywords:** Omicron, Delta, SARS-CoV-2, antibody

## Abstract

In this report, we mechanistically reveal how the Variant of Concern (VOC) SARS-CoV-2 Omicron (B.1.1.529) escapes neutralizing antibody responses, by physio-chemical characterization of this variant in comparison to the wild-type Wuhan and the Delta variant (B.1.617.2). Convalescent sera, as well as sera obtained from participants who received two or three doses of mRNA vaccines (Moderna-mRNA-1273^®^ or Pfizer-BNT162b2^®^), were used for comparison in this study. Our data demonstrate that both Delta, as well as Omicron variants, exhibit a higher affinity for the receptor ACE2, facilitating infection and causing antibody escape by receptor affinity (affinity escape), due to the reduced ability of antibodies to compete with RBD-receptor interaction and virus neutralization. In contrast, only Omicron but not the Delta variant escaped antibody recognition, most likely because only Omicron exhibits the mutation at E484A, a position associated with reduced recognition, resulting in further reduced neutralization (specificity escape). Nevertheless, the immunizations with RNA-based vaccines resulted in marked viral neutralization in vitro for all strains, compatible with the fact that Omicron is still largely susceptible to vaccination-induced antibodies, despite affinity- and specificity escape.

## 1. Introduction

The appearance of the SARS-CoV-2 Omicron variant B.1.1.529 was reported in South Africa (SA) on 24 November 2021 by the World Health Organization (WHO) [1]. Two days later, Omicron was designated as a new variant of concern (VOC) due to its potential to spread rapidly worldwide. The first known sample was collected in SA on 8 November 2021 and the first sample detected outside SA was found in a traveler arriving in Hong Kong from SA via Qatar on 11 November 2021. On 7 January 2022, Omicron was confirmed in 135 different countries worldwide [2].

The WHO has estimated the danger from Omicron as potentially high due to the following reasons: the global risk of perpetuating the SARS-CoV-2 pandemic remains high; recent data have indicated that Omicron shows a significantly higher R-value than the Delta VOC with already enhanced transmission [3,4]. The R-value describes the average number of people that one person will pass on a virus. Increased numbers of cases have previously led to increased hospitalization and mortality, threatening to overwhelm healthcare systems. Preliminary evidence suggested an increased risk of reinfection with Omicron as compared to other VOCs [2]. The concern was also growing because Omicron has spread amongst doubly vaccinated people [5]. In turn, COVID-19 caused by Omicron appeared relatively mild and health care systems have remained functioning despite huge numbers of infected individuals, making it possible that this new variant may help end the pandemic by worldwide spreading and consequent broad immunization with significantly reduced disease severity and mortality.

Until recently, Delta (B.1.617.2) VOC was the predominant SARS-CoV-2 variant before the emergence of Omicron. Delta VOC was discovered in late 2020 in India and had spread to more than 163 nations by 24 August 2021. In June 2021, WHO stated, that the SARS-CoV-2 Delta strain would become the most prevalent strain in the world [1]. Based on recent evidence, Delta strain is 40–60% more transmissible than the previous forms such as wild type (WT) Wuhan, or other recent VOCs such as Alpha (B.1.1.7). Delta has also been associated with an increased risk of hospitalization mostly for unvaccinated or partially vaccinated people [6] which appears different for Omicron. For clarification, WHO has assigned the name Delta variant to lineage B.1.617.2 and it is one of the three Indian variants summarized under B.1.617. The Delta variant B.1.617.2 harbors seven mutations in the spike protein relative to the ancestor Wuhan SARS-CoV-2 strain; two of these mutations (L452R and T478K) are located in the receptor-binding domain (RBD) region [7,8]. Importantly, Delta VOC does not exhibit a mutation at position E484, which is usually associated with a strong reduction of recognition by antibodies [9,10,11].

An unusually large number of thirty-seven mutations has been identified in Omicron in comparison to other VOCs. Most mutations are located in spike protein [5]. Furthermore, fifteen mutations have been detected in RBD region which interacts with the main receptor angiotensin-converting enzyme 2 (ACE2) in comparison to only two mutations in Delta variant [12]. The spike protein, particularly RBD, is the main target for COVID-19 vaccines [13,14]. Computational in silico modeling raised concerns about the possible ability of Omicron to dodge antibody-mediated immunity [15]. Several reasons may account for the reduced ability of antibodies to neutralize emerging VOCs, including specificity alteration of antibody recognition (classical antibody specificity escape) as well as increased RBD-ACE2 affinity that partially outcompetes antibody binding (affinity escape) [9]. So far it seems clear that alteration of antibody recognition of Omicron is related to its E484A mutation [11,16,17,18].

According to WHO, the global impact of Omicron will largely depend on four main aspects:

(1) the transmissibility of the variant,
(2) how well vaccinated and infected people are protected against Omicron,
(3) the virulence of Omicron, and finally
(4) the awareness of the population to take protective measures [2]. In light of the importance of these key parameters, our study aimed at revealing the mechanisms underlying the overserved increased transmissibility and reduced protection by a vaccine- or infection-induced antibodies. Our data demonstrate an increased affinity of Omicron for its receptor ACE2 and reduced recognition by serum antibodies. These factors cause reduced viral neutralization. From a mechanistic point of view, our data demonstrate that Omicron avoids viral neutralization by both classical change of antibody specificity and affinity escape due to the high RBD-ACE2 affinity that outcompetes antibody binding.

## 2. Materials and Methods

### 2.1. Human Sera

Human sera were collected from COVID-19 convalescent patients (infected with wild type Wuhan in the first wave), vaccinated individuals who received two doses of mRNA vaccine (Moderna-mRNA-1273^®^ or Pfizer-BNT162b2^®^), and vaccinated individuals who received a total of three doses of mRNA vaccine (Moderna-mRNA-1273^®^ or Pfizer-BNT162b2^®^). Participants were recruited at the University Hospital of Bern, Bern, Switzerland and Qatar University, Doha, State of Qatar. Sera were collected within one-month post-vaccination [16].

### 2.2. RBD-ACE2 Binding Kinetics

The binding kinetic was performed using Octet RED96E (Sartorius, Göttingen, Germany). SAX sensors were loaded with biotinylated ACE2 25 μg/mL and subsequently quenched with biocytin. RBD was serially diluted in kinetics buffer and dissociation was performed in 300 s. Kinetic buffer (KB) was used to dilute proteins. A loaded sensor run in KB served as a control. The resulting curves were aligned to the beginning of the association, and a 1:1 model was used for global fitting.

### 2.3. RBD Proteins

Recombinant SARS-CoV-2 spike S1 B.1.1.529-Omicron RBD was purchased from (antibodies-online GmbH, Aachen, Germany), recombinant SARS-CoV-2 Spike RBD, and recombinant SARS-CoV-2 Spike RBD B.1.617.2 L452R T478K were purchased from R&D Systems (Minneapolis, MN, USA). Proteins were reconstituted as per the manufacturer’s instructions.

### 2.4. Anti-RBD Titers

ELISA assays were performed as follows; 96 half-well plates were coated overnight with 1 μg/mL of RBD proteins. Plates were blocked for 2 h with 0.15% casein in PBS and bound for 1 h with 1:20 sera diluted in 1:3 steps. Bound IgG antibodies were detected using goat anti-human IgG-POX antibody (Nordic MUbio, Susteren, The Netherlands). ELISA plates were developed using tetramethylbenzidine (TMB) and stopped with 1 M H_2_SO_4_. Absorbance was read at 450 nm and curves were generated using OD_450_ and OD_50_. OD_50_ refers to the measure of the half-max response and OD_450_ refers to an optical density at 450 nm wavelength.

### 2.5. BLI-Based Competitive Assay

The ability of the human sera to compete with ACE2 for binding to RBD_WT_, RBD_Delta,_ and RBD_Omicron_ was tested as previously described by Vogel et al. [11].

### 2.6. Neutralization Assay (Cytopathic Effect-Based Neutralization Assay)

Sera samples were heat-inactivated for 30 min at 56 °C and diluted from 1:20 to 1:320. 100 TCID50 of WT (SARS-CoV-2/ABS/NL20), Delta (SARS-CoV-2/ABSD/NL21), and Omicron (SARS-CoV-2/hCoV-19/NH-RIVM-72291/2021) was added to each well and incubated for 1 h at 37 °C. Following incubation, the mixture was added to a monolayer of Vero cells and incubated for an additional four days at 37 °C. Afterward, wells were inspected for the presence of cytopathic effect (CPE) and titers were expressed as the highest serum dilution which fully inhibits CPE formation (100% inhibition). Sera samples were analyzed as described before [17,19].

### 2.7. Statistics

GraphPad Prism 9.0 (GraphPad Software, Inc, San Diego, CA, USA) was used to perform statistics. *Paired or Unpaired* Student’s *t*-test was performed to test the statistical significance between the two groups (as indicated in the figure’s legend). Statistical significance is displayed as *p* ≤ 0.05 (*), *p* ≤ 0.01 (**), *p* ≤ 0.001 (***), *p* ≤ 0.0001 (****).

## 3. Results

### 3.1. Omicron VOC Accumulated Many More Mutations than Delta Variant

**Figure 1:**
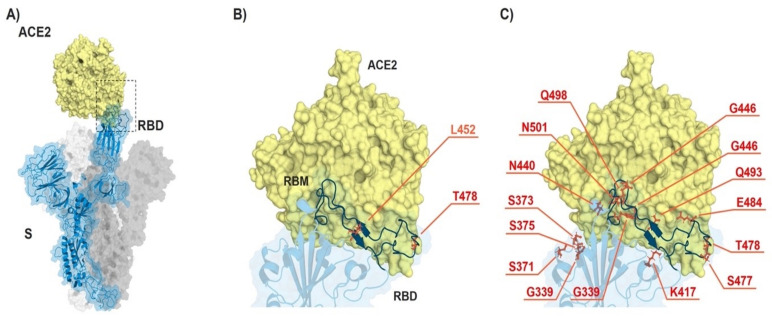
ACE2-spike interaction and mutations found in RBD of B.1.617.2 (Delta) and B.1.1.529 (Omicron). (**A**) S monomer (blue ribbon and surface) bound to ACE2 ectodomain (yellow surface). (**B**) Detail of (**A**), highlighting the mutated residues (orange sticks) in Delta. (**C**) Same as in (**B**) but highlighting the mutated residues in Omicron. From PDB files 6ACG and 2AJF.

The Omicron variant (B.1.1.529) harbors thirty-seven mutations in spike protein, half of which are located in RBD region [20]. In contrast, Delta VOC (B.1.617.2) has only two mutations in its RBD, namely L452R and T478K. L452R mutation has been shown to be common in several newly emerged variants of SARS-CoV-2 including Epsilon, Kappa, Lambda, and Iota. Within RBD region, L452R mutation is located in the receptor-binding motif (RBM) in immediate proximity to ACE2 and has been associated with stronger receptor binding, as well as an immune escape [9,21]. Delta VOC (B.1.617.2) lacks E484Q mutation present in another Indian VOC (Kappa B.1.617.1) but has a unique T478K substitution which is not found in other Indian variants (B.1.617.1 and B.1.617.3) [22]. Interestingly, T478K mutation has also been detected in Omicron VOC. Similar to L452R, T478K is located in RBM domain, making direct contact with ACE2 receptor [22]. Figure 1 and Table 1 show the mutations in RBD region of Omicron (B.1.1.529), Delta (B.1.617.2), and for comparison the ancestor Wuhan. The numerous mutations found in RBD region of Omicron in comparison to Delta suggests that the newly emerged variant may be more immunologically resilient to neutralization by vaccine-induced antibodies.

### 3.2. Binding Kinetics of RBD Omicron (B.1.1.529) to ACE2 Reveal Similar Affinities as RBD Delta (B.1.617.2) VOC

**Figure 2:**
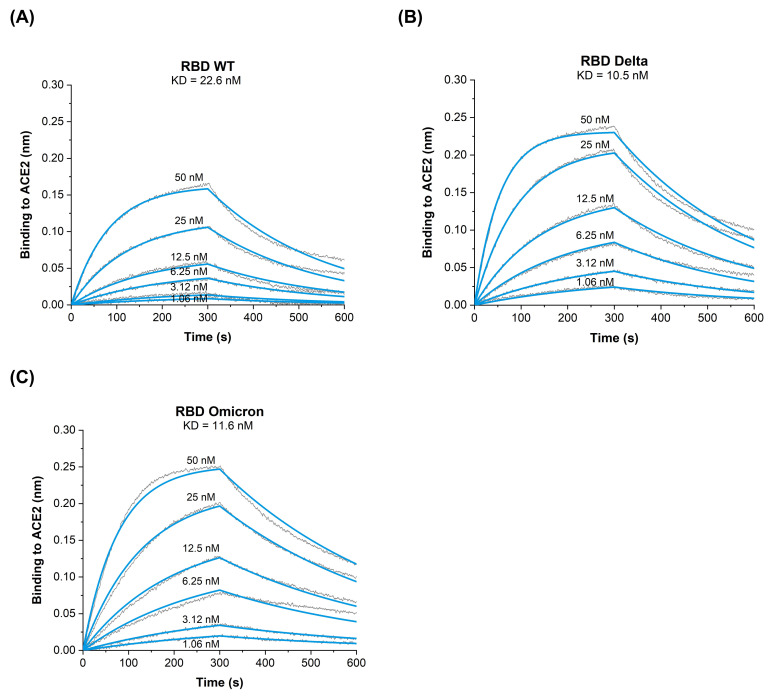
BLI sensograms illustrating ACE2 interactions with (**A**) RBD_WT_; (**B**) RBD_Delta_ (B.1.617.2) and (**C**) RBD_Omicron_ (B.1.1.529). One representative of two similar experiments is shown.

To address the binding properties of RBD_WT_, RBD_Delta,_ and RBD_Omicron_ to human receptor ACE2, we have utilized Biolayer Interferometry (BLI) to measure on- and off-rates as well as the affinity. The results confirm that RBD_WT_ has a binding affinity of K_D_ = 22.6 nM to ACE2 receptor (Figure 2A) which is consistent with our previous findings [9,11]. Interestingly, RBD_Delta_ and RBD_Omicron_ exhibit an approximately similar two-fold increase in the binding affinity to ACE2 (RBD_Delta_ K_D_ = 10.5 nM, Figure 2B) and (RBD_Omicron_ K_D_ = 11.6 nM, Figure 2C). The increased affinity of both RBD_Delta_ and RBD_Omicron_ are caused by an enhanced association rate and decreased dissociation rate (Table 2). These data indicate that RBD_Omicron_ and RBD_Delta_ show increased affinity to ACE2 compared to RBD_WT_. Interestingly, we have previously measured the affinity of RBD of another Indian variant (B.1.1617.1) harboring the two mutations (L452R/E484Q) and found that it was increased another two-fold higher compared to Delta variant ([9] and Table 2). As both Delta and Omicron became world-dominant and thus outcompeted this B.1.1617.1 variant, receptor affinity is apparently just one but not the only parameter that drives the efficiency of viral spread.

### 3.3. Reduced Recognition of Omicron Mutant RBDs by Sera from Convalescent Patients and mRNA Vaccinated Individuals

**Figure 3:**
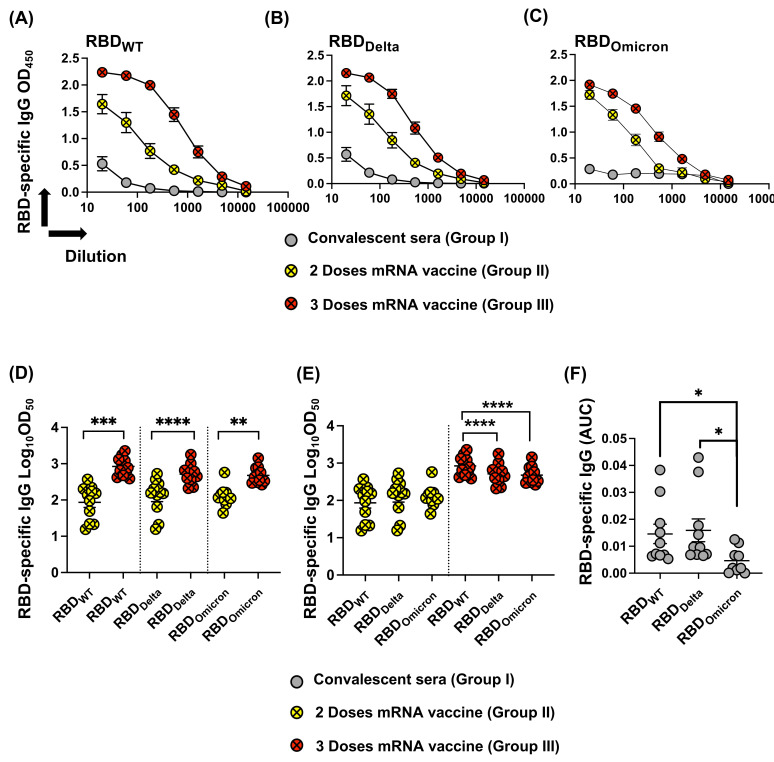
(**A**–**C**) Total IgG to RBD_WT_, RBD_Delta_, and RBD_Omicron_ of sera measured by OD_450_ obtained from COVID-19 convalescent donors (Group I), individuals after two doses of mRNA vaccine (Group II), and individuals after three doses of mRNA vaccine (Group III). (**D**,**E**) Log_10_ OD_50_ of total IgG to RBD_WT_, RBD_Delta_, and RBD_Omicron_, data from (**A**–**C**) (Groups II and III) measured by OD_50_. (**F**) AUC of IgG from convalescent patients to RBD_WT_, RBD_Delta_, and RBD_Omicron_, data from (**A**–**C**) (Group I). Statistical analysis by Student’s *t*-test, (*Unpaired* in (**D**)) and (*Paired* in (**E**,**F**)). *n* = 10–11. One representative of two similar experiments is shown. *p* ≤ 0.05 (*), *p* ≤ 0.01 (**), *p* ≤ 0.001 (***), *p* ≤ 0.0001 (****).

Here, we tested the ability of convalescent sera (Group I), sera from individuals after two doses of mRNA vaccine (Group II), and sera from individuals after three doses of mRNA vaccine (Group III) to recognize RBD_Delta_ and RBD_Omicron_ in comparison to RBD_WT_. Anti-RBD IgG ELISAs revealed that antibodies induced by SARS-CoV-2 infection poorly recognize the different tested mutated RBDs. On the other hand, individuals who received two or three doses of mRNA vaccine could recognize RBD_WT_ equally well as RBD_Delta_ but recognition of RBD_Omicron_ was reduced (Figure 3A–C), see also below.

When measuring the OD_50_ of the induced antibody titer, results indicate, as expected the superiority of the additional booster dose (Group III) (Figure 3D). No statistical difference has been detected in recognizing RBD_WT,_ RBD_Delta_, or RBD_Omicron_ after three doses of mRNA vaccine (Figure 3E). However, a significant reduction in recognizing RBD_Delta_ and RBD_Omicron_ in comparison to RBD_WT_ was noticed after receiving the 3rd dose of the vaccine (*p* < 0.0001) (Figure 3E). Analysis of area under the curve (AUC) for convalescent sera demonstrates strongly impaired recognition of RBD_Omicron_ but not RBD RBD_Delta_ (Figure 3F).

### 3.4. Omicron RBD Resists Neutralization of Receptor Binding by Immune Sera

**Figure 4:**
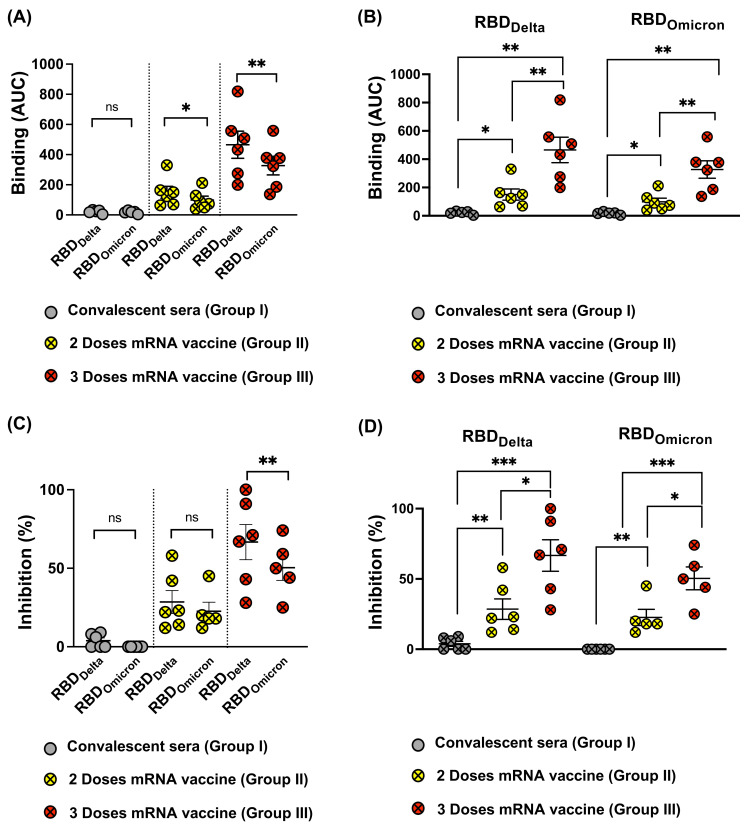
(**A**,**B**) Binding of sera from (Group I, II, and III) to RBD_WT_, RBD_Delta_, and RBD_Omicron_ using BLI, depicted as AUC. (**C**,**D**) Inhibition of RBD_WT_, RBD_Delta_, and RBD_Omicron_ binding to the human receptor ACE2 by 1:80 sera dilution, sera from the three different groups were used in this assay. Statistical analysis by Student’s *t*-test, (*Paired* in (**A**,**C**)) and (*Unpaired* in (**B**,**D**)). *n* = 5–6. One representative of two similar experiments is shown. *p* ≤ 0.05 (*), *p* ≤ 0.01 (**), *p* ≤ 0.001 (***).

To further analyze RBD recognition by the different immune sera (Group I, II, and III), we used Biolayer Interferometry (BLI) to measure antibody binding and the inhibition capability to block receptor interaction of the sera. As suggested by the ELISA results, there was a clear difference between the recognition of RBD_Delta_ and RBD_Omicron,_ with the latter being recognized in a strongly inferior manner. Thus, recognition of the Omicron VOC is further reduced compared to Delta. As seen by ELISA, sera from individuals receiving three mRNA injections reacted best, followed by those receiving two injections. The least reactive were convalescent sera (Figure 4A,B).

Subsequently, we tested the ability of the different sera groups to inhibit RBD_WT_, RBD_Delta_, and RBD_Omicron_ binding to ACE2. Our results indicate that convalescent sera failed to effectively inhibit RBD-ACE2 binding at the concertation tested; such results are consistent with low induced antibody titers observed. Antibodies induced following the 2nd dose of mRNA vaccination, show an average inhibition of 28.5% against RBD_Delta_ versus 27.3% against RBD_Omicron_ with no statistical difference (Figure 4C). The overall average binding inhibition of the two VOCs to ACE2 receptor was dramatically increased after the 3rd dose, accounting for 66% against RBD_Delta_ but only 45% against RBD_Omicron_ (*p* = 0.0011) (Figure 4C). Thus, VOC Omicron evolved furthest to escape neutralization by antibodies induced by RBD_WT_. When comparing the inhibition data following two versus three doses of mRNA vaccine, the results revealed the significant ability of Group III in inhibiting RBD_Delta_ and to a lesser extent RBD_Omicron_ from binding to ACE2 receptor (Figure 4D).

### 3.5. Omicron Resists Viral Neutralization by Immune Sera

**Figure 5:**
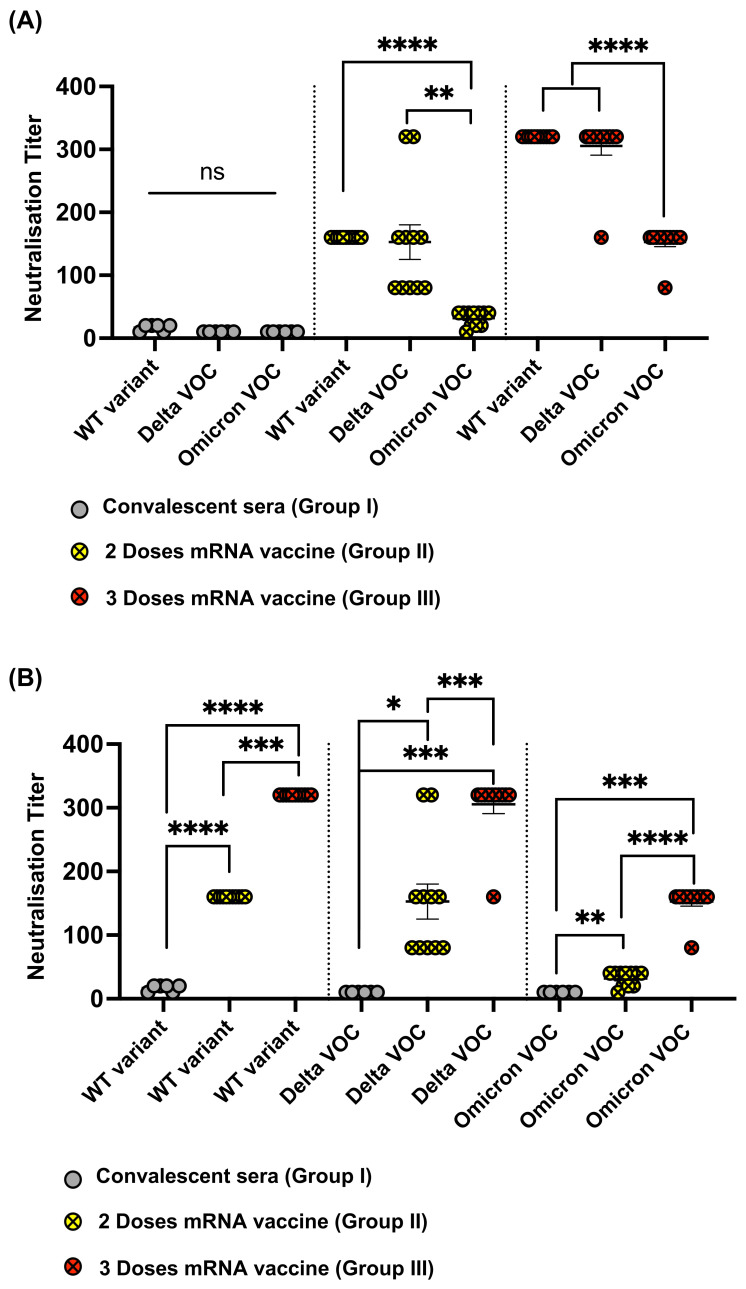
(**A**,**B**) Neutralization titers using CPE assay against WT, Delta, and Omicron VOCs. Sera from Groups I, II, and III were tested. Statistical analysis by Student’s *t*-test, (*Paired* in (**A**)) and (*Unpaired* in (**B**)). *n* = 6 (Group I), *n* = 11 (Groups II and III). *p* ≤ 0.05 (*), *p* ≤ 0.01 (**), *p* ≤ 0.001 (***), *p* ≤ 0.0001 (****).

Cytopathic assay (CPE) was used to assess the neutralization capacity of the different sera Groups (I, II, and III) against WT SARS-CoV-2 as well as Delta and Omicron VOCs. Sera obtained from SARS-CoV-2 infected individuals who received no vaccination showed very low neutralization capacity against WT and both variants tested (Figure 5A). Sera from individuals who received two doses of mRNA vaccine showed similar neutralization titers against both the WT and Delta with no statistical difference (*p* = 0.7961). In contrast to Delta, a significant reduction in the neutralization titer was observed against Omicron in comparison to WT (*p* < 0.0001) and Delta (*p* = 0.0011) (Figure 5A). Such observation also applied to Group III for individuals who received three doses of the vaccine. A significant reduction in sera recognition to Omicron VOC was detected when compared to WT or Delta VOC (*p* < 0.0001).

Next, we analyzed the data with respect to the effect of the vaccination doses on each variant, i.e., a total of two doses vs three doses of mRNA vaccine. As expected, a booster dose of mRNA vaccine caused higher titers than natural infection with COVID-19. A second booster dose (i.e., three doses of mRNA) further enhanced the neutralization titer as shown in Figure 5B. The effect of a total of two or three doses of the vaccine was more variable and overall lower against Delta VOC than what was observed against the WT. Most importantly and as explained earlier, the overall neutralization titer against Omicron was reduced the most compared to the other variants (Figure 5B).

## 4. Discussion

In this report, we have immunologically and mechanistically assessed the difference between Delta VOC (B.1.617.2) and Omicron (B.1.1.529) VOC using three different groups of sera. Omicron harbors fifteen mutations in RBD region besides other mutations in its spike protein. In contrast, Delta has only two critical mutations, L452R and T478K. The latter mutation has also been detected in Omicron. Both L452R and T478K mutations are located in RBD, the essential region for viral binding to ACE2 and entry into receptor cells. The exact function of the remaining mutations in Omicron has yet to be revealed; nevertheless, the unusually large number of mutations in Omicron raised serious concerns about the reduced efficacy of the licensed COVID-19 vaccines to induce Omicron-neutralizing antibodies.

Recent studies have assessed the binding affinity of RBD_Omicron_ to human ACE2 receptors in comparison to the ancestral Wuhan strain, via atomistic molecular dynamics simulation [23] or surface plasmon resonance [5]. The results revealed stronger binding to human ACE2 receptor, indicating that Omicron infects cells by a similar mechanism to the WT (via ACE2) and suggesting that the clinically observed increased infectivity might be due to stronger binding interaction with ACE2. By performing Biolayer Interferometry (BLI) we demonstrate here definitively at the biophysical level that both RBD_Omicron_, as well as RBD_Delta_ variant, have a well-defined two-fold increased affinity to ACE2 compared to RBD_wt_.

Our data show that Omicron has developed two mechanisms that account for the escape from neutralizing antibodies: First, RBD mutations result in a partial mismatch with the initial antibody response, such that antibodies generated by RBDs from previous infection or vaccination are less well-blocking due to their specificity for previous RBD versions. Particularly, E484A mutation accounts for this type of specificity-escape, as it prominently changes an important epitope for neutralizing antibodies. Second, Omicron’s increased RBD-ACE2 affinity represents a challenge for neutralizing antibodies, as the virus’ RBD binds so strongly to ACE2 that antibodies become unable to compete and thus cannot block the virus. We call this phenomenon ‘affinity escape’ as this type of immune escape is clearly different from the more classical antibody specificity escape.

Our findings are consistent with the fact that Coronaviruses do not form serotypes, in contrast -for example- to Polio and Dengue viruses [24]. Coronaviruses are large viruses with about three times more RNA nucleotides than most other RNA viruses. Despite the many small mutations that accumulate over time, Coronaviruses depend on a relatively high degree of genetic and structural stability which is assured by the RNA proofreading system of Coronaviruses [25]. For enhancing transmission, an apparently better strategy than novel serotypes is to increase receptor affinity, a phenomenon that is now well documented for SARS-CoV-2 [9,11,26,27]. The new phenomenon of ‘affinity escape’ from neutralizing antibodies allows for a better understanding of the interactions of the virus with the immune system. We suggest that vaccines may be rendered more efficient by optimization for inducing large numbers of neutralizing antibodies with high affinity. It is well known that booster vaccinations significantly contribute to this aim, as continued affinity maturation in germinal centers of secondary lymphoid organs selectively generates and promotes high-affinity antibodies [28]. Indeed, individuals who have received three rather than only two RNA vaccinations reach relatively high protection from Omicron [29,30,31], although these vaccinations are still based on RBD_WT_ (Wuhan), supporting the notion that antibody specificity may not be the major reason for a breakthrough in infections. Overall, the net effect of the antibody response is a combination of specificity, activity, and titer. Mass action effect (i.e., a high magnitude response) can compensate for some shortcomings in affinity.

In summary, both Delta and Omicron VOCs show enhanced receptor affinity translated into reduced neutralization (affinity escape). For Omicron, reduction in a neutralization is, however, much more pronounced as its affinity escape is combined with specificity escape.

## Figures and Tables

**Table 1:** Mutations in RBD in Delta (B.1.617.2) and Omicron (B.1.1.529) VOC.

RBD_Delta_ (B.1.617.2)	RBD_Omicron_ (B.1.1.529)
L452R and **T478K**	K417N, N440K, G446S, S477N, **T478K**, E484A, Q493R, G496S, Q498R, N501Y, and Y505H

Identical mutation sites in both Delta and Omicron variants are shown in magenta.

**Table 2:** Kinetic parameters for RBD-ACE2 interaction calculated by BLI.

Analyte	K_D_ [M]	k_on_ [M^−1^s^−1^]	k_off_ [s^−1^]
RBD_WT_	22.6 × 10^−9^	1.7 × 10^5^	3.9 × 10^−3^
RBD_Delta_	10.5 × 10^−9^	3.1 × 10^5^	2.3 × 10^−3^
RBD_Omicron_	11.6 × 10^−9^	2.1 × 10^5^	2.5 × 10^−3^
RBD_L452R/E484Q_ [9]	4.6 × 10^−9^	7.2 × 10^5^	3.3 × 10^−3^

## Data Availability

The datasets generated during and/or analyzed during the current study are available from the corresponding author on reasonable request.
